# Altered Gut Microbiota in Chinese Children With Autism Spectrum Disorders

**DOI:** 10.3389/fcimb.2019.00040

**Published:** 2019-03-06

**Authors:** Bingjie Ma, Jingjing Liang, Meixia Dai, Jue Wang, Jingyin Luo, Zheqing Zhang, Jin Jing

**Affiliations:** ^1^Department of Maternal and Child Health, School of Public Health, Sun Yat-sen University, Guangzhou, China; ^2^Department of Child Health Care, Guangzhou Women and Children's Medical Center, Guangzhou Medical University, Guangzhou, China; ^3^Guangdong Provincial Key Laboratory of Tropical Disease Research, Department of Nutrition and Food Hygiene, School of Public Health, Southern Medical University, Guangzhou, China

**Keywords:** autism spectrum disorder, gut microbiota, 16S rRNA, Chinese children, case control

## Abstract

The link between gut microbes and autism spectrum disorders (ASD) has been already observed in some studies, but some bacterial families/species were found to be inconsistently up or down regulated. This issue has been rarely explored in the Chinese population. In this study, we assessed whether or not gut microbiota dysbiosis was associated with children with ASD in China. We enrolled 45 children with ASD (6–9 years of age; 39 boys and 6 girls) and 45 sex- and age-matched neurotypical children. Dietary and other socio-demographic information was obtained via questionnaires. We characterized the composition of the fecal microbiota using bacterial 16S ribosomal RNA (16S rRNA) gene sequencing. The ASD group showed less diversity and richness of gut microbiota than the neurotypical group, as estimated by the abundance-based coverage estimator index and the phylogenetic diversity index. The analysis of beta diversity showed an altered microbial community structure in the ASD group. After adjustment for confounders and multiple testing corrections, no significant group difference was found in the relative abundance of microbiota on the level of the phylum. At the family level, children with ASD had a lower relative abundance of *Acidaminococcaceae* than the healthy controls. Moreover, a decrease in the relative abundance of genera *Lachnoclostridium, Tyzzerella subgroup 4, Flavonifractor*, and *unidentified Lachnospiraceae* was observed in ASD group. This study provides further evidence of intestinal microbial dysbiosis in ASD and sheds light on the characteristics of the gut microbiome of autistic children in China.

## Introduction

Autism spectrum disorders (ASD) are complex neurodevelopmental disorders characterized by impairment of social interaction and communication and restricted, repetitive behavior (American Psychiatric Association, 2013). The origin of ASD is not fully understood, and evidence suggests that both genetic and environmental factors are involved (Mcelhanon et al., 2014; Colvert et al., 2015). In addition to the core symptoms, evidence shows that gastrointestinal (GI) symptoms, including constipation, abdominal pain, diarrhea and gaseousness and vomiting, are also prominent in individuals with ASD, with estimates ranging from 9 to 70% (Buie et al., 2010). Furthermore, several studies have reported a strong positive correlation between GI problems and the severity of ASD (Buie et al., 2010; Adams et al., 2011; Tomova et al., 2015). The GI microbiota is an integral part of human physiology. It influences brain development and behavior through the neuroendocrine, neuroimmune and autonomic nervous systems (Ding et al., 2017). Investigators have highlighted the existence of a so-called “microbiota-gut-brain axis,” which supports the hypothesis that the gut microbiota could trigger neuropsychiatric symptoms in subjects with ASD (Sampson and Mazmanian, 2015; Kraneveld et al., 2016; Sharon et al., 2016).

The gut microbiota–ASD connection has been tested in animal models of ASD, and the microbiota was mechanistically linked to abnormal metabolites and behavior (Hsiao et al., 2013; Mayer et al., 2014; Arentsen et al., 2015). Various bacterial species have been shown to be involved in microbial dysbiosis in children with ASD (Finegold et al., 2010; Martirosian et al., 2011; Williams et al., 2011; Kang et al., 2013; De Angelis et al., 2015; Strati et al., 2017; Zhang et al., 2018; Liu et al., 2019). Specifically, a higher bacterial incidence of potentially harmful *Clostridium* clusters was observed in autistic children than in healthy controls (Argou-Cardozo and Zeidan-Chulia, 2018). Beneficial bacteria (i.e., *Bifidobacterium, Lactobacillus*) were reported to be inconsistently up or down regulated in different studies (Wang et al., 2011; Tomova et al., 2015). There is also no established consensus on observations on individual bacterial taxa. For example, *Desulfovibrio* and *Akkermansia* levels in autistic children were shown to be either higher (De Angelis et al., 2015) or lower (Wang et al., 2011; Kang et al., 2013). Although some studies supported a reduction of the *Bacteroidetes*/*Firmicutes* ratio in children with ASD (Williams et al., 2011; Tomova et al., 2015; Strati et al., 2017), this was incongruent with other findings (De Angelis et al., 2015; Liu et al., 2019). Some human epidemiologic studies focusing on children with ASD and their neurotypical siblings reported either no difference (Gondalia et al., 2012; Son et al., 2015) or an aberrant composition of gut microbiota (De Angelis et al., 2015). It should be noted that neurotypical siblings, however, may differ from the general neurotypical population.

Microbiota varies with age, genetics, dietary habits, geographic environment, and other host-associated factors (Yatsunenko et al., 2012). Recent studies have also shown substantial divergence in the microbiome structure between individuals from different races and ethnicities (Chong et al., 2015; Gupta et al., 2017). To the best of our knowledge, only two studies have addressed this topic in the context of ASD in the Chinese population (Zhang et al., 2018; Liu et al., 2019). Therefore, we studied the bacterial gut microbiota between 45 children with ASD between 6 and 9 years of age and 45 sex- and age-matched neurotypical children by sequencing the V3/V4 regions of the 16S rRNA from fecal samples.

## Methods

### Subject Recruitment

Between December 2015 and July 2017, 45 children with ASD between 6 and 9 years of age (39 males and 6 females) were enrolled in the Center for Child and Adolescent Psychology and Behavioral Development of Sun Yat-sen University in Guangzhou, China. The children received a diagnosis of ASD and met the criteria in the Diagnostic and Statistical Manual of Mental Disorders, 5th Edition (DMS-5) (American Psychiatric Association, 2013). Two experienced developmental and behavioral pediatricians further confirmed the diagnosis with the Childhood Autism Rating Scale (CARS) (Schopler et al., 1980). Children with ASD were excluded from the study if they had a history of Rett syndrome, cerebral palsy, other congenital diseases, and acute or chronic affective diseases in the past 3 months. Sex- and age-matched healthy, developmentally normal children, who were unrelated to the autistic individuals, were recruited from primary schools and sent an invitation letter. The participants did not receive antibiotic treatment, probiotics, prebiotics or any other medical treatment that could influence the intestinal microbiota during the 3 months before they were enrolled in the study.

### Anthropometric Measurements

We measured weight to the nearest 0.1 kg, with the participants wearing light clothes and no shoes, and height to the nearest 0.1 cm, with the child in an upright position.

### Sample Collection and DNA Extraction

Stool specimens were collected from 39 out of 45 children with ASD and all the controls during the process of physical and mental examination. The specimens were frozen at −80°C within 10 min and stored until DNA extraction. In the remaining six ASD cases, the parents were asked to collect the stool samples at home and place them in a sterile plastic container. The samples were refrigerated at home and transported to the research facility within 12 h in a cooler with ice packs. Following the manufacturer's instructions, the fecal microbial DNA was extracted from 250 mg of feces using QIAamp Fast DNA Stool Mini kit (Qiagen, Hilden, Germany). The DNA concentration and purity were monitored on 1% agarose gels. According to the concentration, the DNA was diluted to 1 ng/μL using sterile water and stored at −20°C before analysis.

### 16S rRNA Sequencing Analysis

For each DNA sample, we amplified the bacterial 16S rRNA genes using a primer set specific for V3–V4 hypervariable regions (341F: CCT AYG GGR BGC ASC AG, 806R: GGA CTA CNN GGG TAT CTA AT) (Xiao et al., 2016) with a unique barcode for multiplexing. The PCR reactions were carried out in 30-μl reactions with 15 μl of Phusion High-Fidelity PCR Master Mix (New England Biolabs), 0.2 pmol/μl of forward and reverse primers, and about 10 ng of DNA template. Thermal cycling consisted of initial denaturation at 98°C for 1 min, 30 cycles of 98°C for 10 s, annealing at 50°C for 30 s, elongation at 72°C for 1 min, and finally an extension of 72°C for 5 min. The amplified products were then checked, purified and quantified, according to the respective manufacturers' instructions. Following the manufacturer's recommendations, sequencing libraries were generated using the TruSeq DNA PCR-Free Sample Preparation Kit (Illumina, USA) and index codes were added. The library quality was assessed on the Qubit@ 2.0 Fluorometer (Thermo Scientific) and Agilent Bioanalyzer 2100 system. Finally, 250 bp paired-end reads were obtained using the IlluminaHiSeq2500 platform. The paired-end reads from the original DNA fragments were merged using FLASH (V1.2.7, http://ccb.jhu.edu/software/FLASH/) (Magoc and Salzberg, 2011). We assigned paired-end reads to each sample according to their unique barcodes. The tags were compared with the reference database of Broad Microbiome Utilities (“Gold database,” version 20110519, http://drive5.com/uchime/uchime_download.html) using UCHIME algorithm (UCHIME Algorithm, http://www.drive5.com/usearch/manual/uchime_algo.html) (Edgar et al., 2011) to detect chimera sequences; putative chimeric sequences were removed. OTU grouping was performed using the Uparse software package (Uparse v7.0.1001, http://drive5.com/uparse/) (Edgar, 2013). Sequences with >97% similarity were assigned to the same operational taxonomic units (OTUs). For each representative sequence, Mothur classifier (with a cut-off value of 0.8) was used to annotate taxonomic information against SILVA Database (version 128, https://www.arb-silva.de/) (Quast et al., 2013). In addition, the MUSCLE software (Version 3.8.31, http://www.drive5.com/muscle/) (Edgar, 2004) was used to estimate the phylogenetic tree to be used in the Unifrac distances calculation. The functional potential of gut microbiota was inferred with Tax4Fun (Asshauer et al., 2015) using the SILVA database as a reference. Further, we obtained a prediction of the Kyoto Encyclopedia of Genes and Genomes (KEGG) ortholog (KO) functional profiles.

### Covariates

A validated 79-item food frequency questionnaire was used to assess the usual dietary intake for the past year (Zhang and Ho, 2009). The parents and their children were asked to complete the questionnaire together. Photographs of food portion sizes were provided to help estimate the amount of food consumed. For each food item, five possible frequencies (never, per year, per month, per week, and per day) and a quantitative (amount) response were available. Daily mean nutrient and energy intakes were calculated using the Chinese Food Composition Table, 2009 (Yang et al., 2009). In addition, individual daily intake of each nutrient was adjusted for total energy intake by using the regression residual method (Willett et al., 1997). Trained interviewers conducted face-to-face interviews to collect essential information on gestational age, delivery mode, birth weight and height of children, feeding patterns, and parent educational level. We classified the parent educational level into four categories: primary or less, secondary, graduate, or postgraduate or above.

### Statistical Analyses

Continuous variables were presented as mean ± standard deviation, and comparisons between ASD and neurotypical groups were performed with paired Student *t*-test or Wilcoxon matched pairs test. Categorical variables were presented as proportions, and the groups were compared using chi-square tests. QIIME software (Version 1.7.0) (Caporaso et al., 2010) was used to calculate the alpha and beta diversity estimates, which were displayed by R software (Version 2.15.3). The indicators of alpha diversity included abundance-based coverage estimator (ACE), Chao1, Shannon, and the phylogenetic diversity (PD) index. The difference in the alpha diversity between the groups was tested with a paired Student *t*-test. The principal coordinates analysis (PCoA) using Bray-Curtis dissimilarity and unweighted and weighted UniFrac distances were performed to assess the beta diversity of the bacterial community. The permutational multivariate analysis of variance (PERMANOVA) analysis was conducted using the adonis function of the vegan package in R with 999 permutations (Oksanen et al., 2013). The differences of the relative abundance between the groups at each taxonomic level (phylum, class, order, family, genus, and species) and the group differences in the functional category abundances were analyzed using paired Student *t*-test. The false discovery rate (FDR) was used for the *P*-value correction upon multiple comparisons, using the Benjamini-Hochberg method (Benjamini and Hochberg, 1995). In addition, multivariable models were adjusted for gestational age, delivery mode, parent educational levels, and daily intakes of total energy, protein, fat, carbohydrates, and fiber.

## Results

### Characteristics of Study Participants

We recruited 45 subjects with ASD (average age 7.04 ± 1.19 years; male: female, 39:6) and 45 sex- and age-matched neurotypical controls. Table 1 shows the characteristics of the subjects. We observed a significant difference in the distribution of paternal educational level between the two groups (*P* = 0.020). No significant differences were found in the other characteristics between the ASD and neurotypical groups.

**Table 1:** Characteristics of study participants.

**Variables**	**NT group**	**ASD group**	***P*-value**
Sample size	45	45	
Age, year	7.27 ± 1.07	7.04 ± 1.19	0.375
Sex (%,n)			0.765
Male	86.67% (39)	86.67% (39)	
Female	13.33% (6)	13.33% (6)	
Height, cm	126.86 ± 7.13	126.33 ± 7.65	0.620
Weight, kg	26.44 ± 7.17	26.66 ± 6.80	0.840
Birth length, cm	50.93 ± 2.42	50.55 ± 2.86	0.529
Birth weight, kg	3.24 ± 0.36	3.10 ± 0.42	0.059
Gestational age (%,n)			0.494
Term	100% (45)	95.56% (43)	
Preterm	0 (0)	4.44% (2)	
Delivery mode(%,n)			0.833
Cesarean section	51.11% (23)	55.56% (25)	
Natural birth	48.89% (22)	44.44% (20)	
Feeding patterns (%,n)			0.793
Breastfeeding	82.22% (37)	77.78% (35)	
Artificial feeding	17.78% (8)	22.22% (10)	
Paternal educational level (%,n)			**0.020**
Primary or less	24.44% (11)	13.33% (6)	
Secondary	8.89% (4)	33.33% (15)	
University	57.78%(26)	51.11% (23)	
Post graduate or above	8.89%(4)	2.22% (1)	
Maternal educational level(%,n)			0.503
Primary or less	13.33% (6)	6.67% (3)	
Secondary	22.22% (10)	31.11% (14)	
University	57.78%(26)	51.11% (23)	
Post graduate or above	6.67%(3)	11.11% (5)	
Dietary consumption			
Total energy (kcal/d)	1603.77 ± 484.52	1418.30 ± 472.58	0.064
Protein ^a^(g/d)	70.78 ± 12.19	67.32 ± 10.46	0.172
Fat ^a^(g/d)	43.57 ± 11.04	48.43 ± 10.40	0.076
Carbohydrate ^a^(g/d)	213.94 ± 30.68	206.28 ± 26.49	0.273
Fiber ^a^(g/d)	8.48 ± 2.85	8.07 ± 2.46	0.481
CARS	NA	36.31 ± 6.32	/

*Data are expressed as mean ± standard deviation when applicable. ASD, autism spectrum disorders; NT, neurotypical; CARS, Childhood Autism Rating Scale; NA, not applicable. The P-value was compared between ASD and NT groups. Bold indicates P < 0.05*.

a *Energy-adjusted by the residual method*.

### Differences of Gut Microbial Diversity Between ASD and Healthy Children

**Figure 1:**
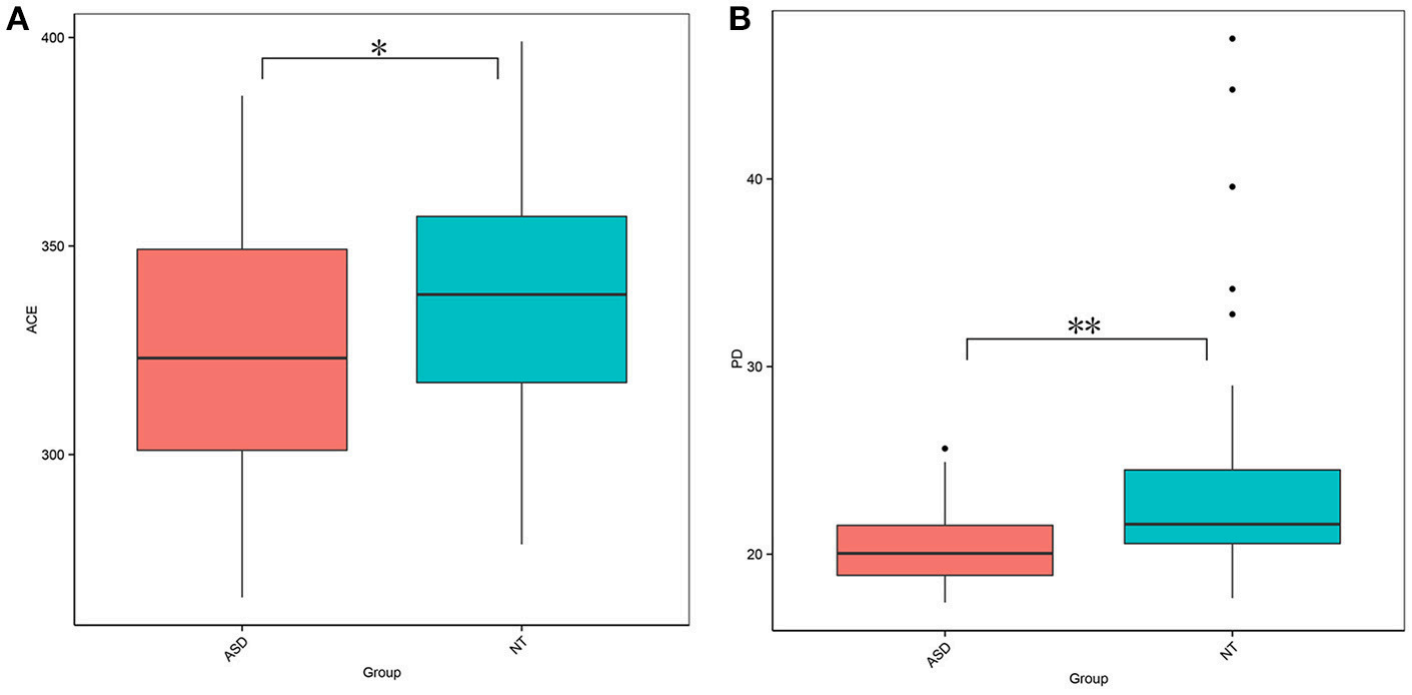
Comparison on bacterial richness and diversity between NT and ASD groups. Comparison of **(A)** ACE estimator and **(B)** PD index between ASD (red-colored box) and NT (blue-colored box) groups (^*^P < 0.05, ^**^P < 0.01 by paired Student *t*-test). ASD, autism spectrum disorders; NT, neurotypical.

This study obtained 5,903,119 reads of high quality and classification at an average of 65,590 reads per sample (Supplemental Table S1). At a 97% similarity level, this study identified 30,217 OTUs in all samples and an average of 336 OTUs per sample (Supplemental Table S1). The ASD group had a lower biodiversity than the neurotypical group, as indicated by the ACE estimator (329.27 ± 30.52 vs. 343.93 ± 32.45, *P* = 0.040; Figure 1A and Supplemental Table S2) and PD index (19.20 ± 1.98 vs. 22.07 ± 5.64, *P* = 0.002; Figure 1B and Supplemental Table S2).

**Figure 2:**
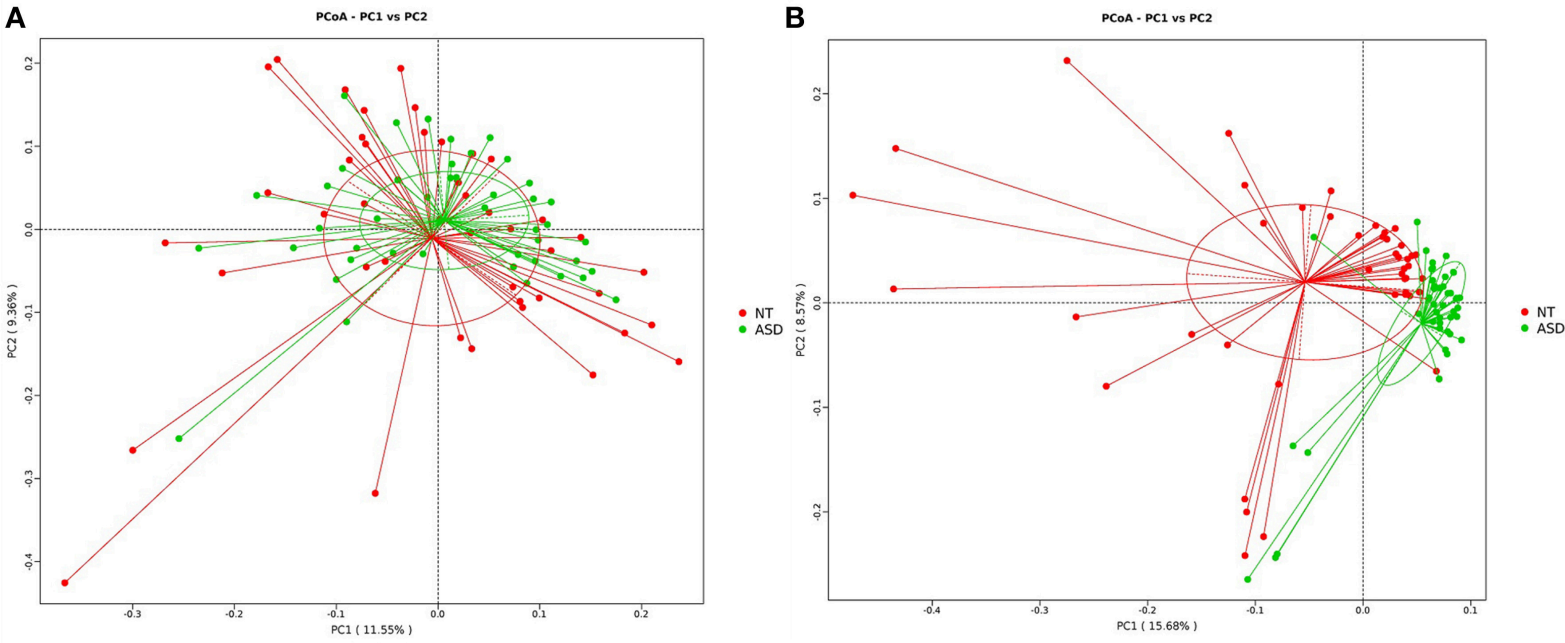
PCoA of bacterial beta diversity based on **(A)** Bray-Curtis dissimilarity **(B)** unweighted UniFrac distances. Subjects with ASD and NT subjects are colored in green and red, respectively. ASD, autism spectrum disorders; NT, neurotypical.

The PCoA analysis calculated on the Bray-Curtis dissimilarity and unweighted UniFrac distances revealed that the gut microbiota of the subjects with ASD clustered apart from that of neurotypical subjects (*P* = 0.002, *P* = 0.001, respectively; Figure 2 and Supplemental Table S3). The PCoA analysis of the weighted UniFrac distances showed no group difference (*P* = 0.128, Supplemental Table S3).

### Differences of Microbiota Comparison Between ASD and Healthy Children

The analysis of the microbial composition of the ASD and control groups at the phylum level (Supplemental Table S4 and Supplemental Figure S1) showed that four phyla, including *Firmicutes, Bacteroidetes, Actinobacteria*, and *Proteobacteria*, made up the main part of the gut microbiota. However, we observed no significant difference in terms of the microbial composition at the phylum level between the two groups. The ratio of *Firmicutes/Bacteroidetes* was also not significantly different between the ASD and healthy children (1.39 vs. 1.82, *P* = 0.108). FDR-adjusted differences at the family level did not reach statistical significance except for *Acidaminococcaceae* (0.44 and 0.16% for healthy controls and ASD children, respectively; P_FDR_ = 0.029; Table 2). No significant group difference was found in the relative abundance of microbiota at the class and order level (Supplemental Tables S5, S6).

**Table 2:** Relative abundance of top 10 abundant families detected in NT and ASD groups and family presenting significant difference between NT and ASD groups.

**Family**	**Mean** **±** **SD**		**Paired student *t*-test**	Adjusted P^*^	**FDR-corrected *P***
	**ASD group**	**NT group**	***P***		
*Bacteroidaceae*	31.57, 8.27	27.95, 11.84	0.105	0.421	0.532
*Prevotellaceae*	3.78, 7.16	4.64, 11.51	0.679	0.854	0.891
*Clostridia Clostridiales Lachnospiraceae*	23.69, 5.87	25.84, 8.52	0.162	0.160	0.361
*Ruminococcaceae*	22.34, 7.28	20.40, 6.67	0.245	0.079	0.285
*Fusobacteriaceae*	2.26, 6.79	1.03, 2.08	0.203	0.314	0.443
*Veillonellaceae*	0.94, 1.36	3.00, 6.33	**0.021**	0.107	0.309
*Bifidobacteriaceae*	3.07, 3.67	3.00, 2.91	0.925	0.791	0.837
*Porphyromonadaceae*	2.15, 1.16	2.70, 2.62	0.223	0.224	0.424
*Unidentified Firmicutes Clostridiales Lachnospiraceae*	1.22, 1.18	1.49, 2.16	0.497	0.415	0.532
*Rikenellaceae*	2.23, 1.92	2.26, 2.27	0.960	0.606	0.773
*Veillonellaceae*	0.94, 1.36	3.00, 6.33	**0.021**	0.107	0.309
*Pasteurellaceae*	0.65, 1.31	0.21, 0.42	**0.038**	0.064	0.257
*Enterobacteriaceae*	0.29, 0.29	0.78, 1.14	**0.007**	**0.036**	0.309
*Acidaminococcaceae*	0.16, 0.15	0.44, 0.48	**0.001**	**0.002**	**0.029**
*Desulfovibrionaceae*	0.09, 0.09	0.15, 0.14	**0.040**	**0.036**	0.201
*Lactobacillaceae*	0.01, 0.01	0.05, 0.12	**0.024**	**0.036**	0.201

ASD, autism spectrum disorders; NT, neurotypical. Relative abundance is shown as mean values (%) ± standard deviation (SD). P^*^ value was adjusted for gestational age, delivery mode, parent educational levels, total energy, protein, fat, carbohydrate and fiber. Bold indicates P < 0.05.

At the genera level, *Bacteroides* constituted the most abundant genus in both the ASD and healthy control groups, but with no significant difference between the two groups (31.59 vs. 27.98%, P_FDR_ = 0.600; Table 3 and Supplemental Figure S2). Compared to the neurotypical controls after adjustment for covariates and multiple comparison correction, *Lachnoclostridium* (3.55 vs. 2.25%, P_FDR_ = 0.005), *Tyzzerella subgroup 4* (0.50 vs. 0.03%, P_FDR_ = 0.002), *Flavonifractor* (0.16 vs. 0.08%, P_FDR_ = 0.002) and *unidentified Lachnospiraceae* (0.13 vs. 0.06%, P_FDR_ = 0.002) were less abundant in the ASD group (Table 3). At the species level, *Clostridium clostridioforme* was more abundant in the ASD group (0.10 vs. 0.22%, P_FDR_ = 0.005; Supplemental Table S7).

**Table 3:** Relative abundance of top 10 abundant genera detected in NT and ASD groups and genera presenting significant difference between NT and ASD groups.

**Genera**	**Mean** **±** **SD**		**Paired Student *t*-test**	Adjusted P^*^	**FDR-corrected *P***
	**ASD group**	**NT group**	***P***		
*Bacteroides*	31.59, 8.28	27.98, 11.84	0.105	0.421	0.600
*Prevotella subgroup 9*	2.71, 6.54	4.42, 11.51	0.396	0.594	0.713
*Fusobacterium*	2.26, 6.79	1.03, 2.08	0.201	0.312	0.492
*Megamonas*	0.55, 0.99	2.51, 6.33	**0.037**	0.162	0.373
*Faecalibacterium*	13.99, 4.92	12.49, 5.23	0.225	0.081	0.294
*Alloprevotella*	0.28, 0.68	0.06, 0.19	**0.048**	0.059	0.249
*Bifidobacterium*	3.07, 3.67	3.00, 2.91	0.925	0.791	0.856
*Pseudobutyrivibrio*	4.63, 2.66	4.36, 3.57	0.623	0.453	0.622
*Parabacteroides*	1.64, 0.96	2.02, 2.31	0.312	0.288	0.477
*Prevotellaceae NK3B31 group*	0.30, 1.68	0.04, 0.13	0.297	0.443	0.612
*Megamonas*	0.55, 0.99	2.51, 6.33	**0.037**	0.162	0.373
*Alloprevotella*	0.28, 0.68	0.06, 0.19	**0.048**	0.059	0.249
*Lachnoclostridium*	2.25, 0.72	3.55, 1.92	**2.85** **×** **10**^**−4**^	**1.54** **×** **10**^**−4**^	**0.005**
*Haemophilus*	0.66, 1.31	0.22, 0.42	**0.037**	0.064	0.252
*Escherichia-Shigella*	0.18, 0.25	0.53, 0.81	**0.010**	**0.043**	0.216
*Ruminiclostridium subgroup 6*	0.01, 0.02	0.19, 0.61	**0.050**	0.092	0.317
*Prevotella subgroup 2*	0.26, 0.76	0.02, 0.04	**0.045**	0.109	0.324
*Lachnospiraceae UCG-004*	0.63, 0.51	0.44, 0.23	**0.027**	**0.023**	0.151
*Tyzzerella subgroup 4*	0.13, 0.08	0.50, 0.51	**2.99** **×** **10**^**−5**^	**3.74** **×** **10**^**−5**^	**0.002**
*Phascolarctobacterium*	0.16, 0.15	0.36, 0.42	**0.007**	**0.014**	0.123
*Erysipelatoclostridium*	0.04, 0.05	0.20, 0.37	**0.009**	**0.004**	0.067
*Unidentified Ruminococcaceae*	0.10, 0.06	0.18, 0.22	**0.012**	0.063	0.252
*Eggerthella*	0.02, 0.01	0.07, 0.13	**0.017**	**0.013**	0.123
*Coprococcus subgroup 3*	0.14, 0.09	0.21, 0.20	**0.027**	0.101	0.324
*Odoribacter*	0.12, 0.12	0.21, 0.17	**0.009**	**0.009**	0.111
*Lactobacillus*	0.01, 0.01	0.05, 0.13	**0.024**	**0.036**	0.196
*Flavonifractor*	0.08, 0.03	0.16, 0.11	**7.14 × 10**^**−6**^	**2.75** **×** **10**^**−5**^	**0.002**
*Ruminococcaceae UCG-010*	0.06, 0.09	0.03, 0.05	**0.048**	**0.020**	0.151
*Unidentified Lachnospiraceae*	0.06, 0.03	0.13, 0.11	**4.14** **×** **10**^**−5**^	**6.12** **×** **10**^**−5**^	**0.002**
*Lachnospiraceae FCS020 group*	0.12, 0.06	0.08, 0.06	**4.26** **×** **10**^**−4**^	**0.005**	0.070
*Bilophila*	0.05, 0.04	0.09, 0.06	**0.003**	**0.004**	0.057

ASD, autism spectrum disorders; NT, neurotypical. Relative abundance is shown as mean values (%), standard deviation (SD). P^*^ value was adjusted for gestational age, delivery mode, parent educational levels, total energy, protein, fat, carbohydrate and fiber. Bold indicates P < 0.05.

### Functional Capability Analysis

**Figure 3:**
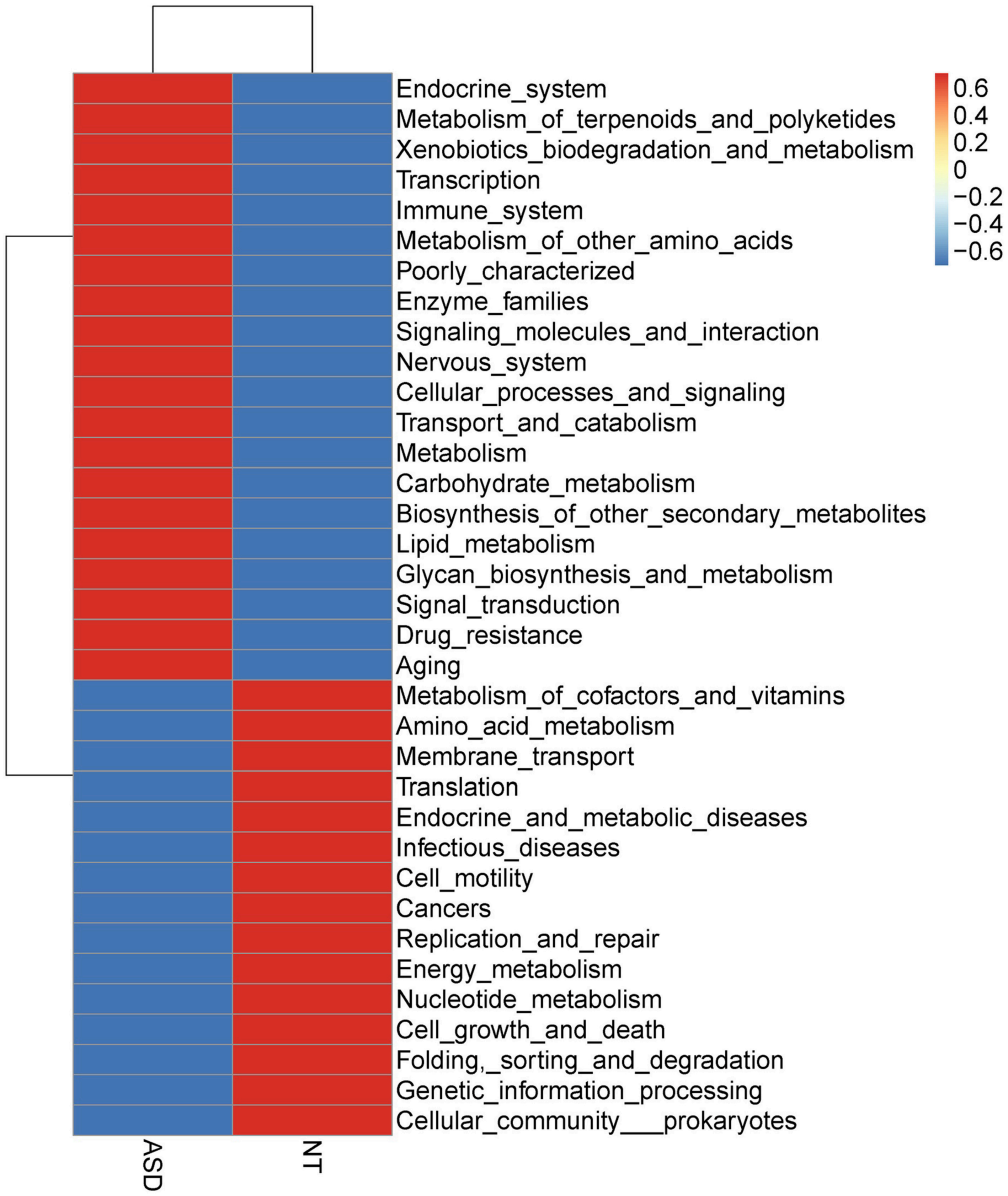
Heatmap based on mean abundances of level 2 KEGG pathways between ASD and NT groups. ASD, autism spectrum disorders; NT, neurotypical.

The overall functional structure of ASD group was dominated by 20 functions, such as *Cellular processes and signaling, Metabolism*, and *Carbohydrate metabolism*, while the neurotypical group was dominated by the other 15 functions (Figure 3). However, no significant functional differences were found between groups after FDR correction (Supplemental Table S8).

## Discussion

Based on the 16S rRNA data from 45 autistic cases and 45 sex- and age-matched controls, we describe the differences in the gut microbiota features in Chinese children. The children with ASD displayed a less diverse gut microbiome than the neurotypical controls. Children with ASD exhibited a lower relative abundance of *Acidaminococcaceae* than the healthy controls at the family level. A decrease in the relative abundance of genera *Lachnoclostridium, Tyzzerella subgroup 4, Flavonifractor*, and *unidentified Lachnospiraceae* was also found in the ASD group than in the neurotypical children.

### Diversity of Microbiota

In our data, the analysis of beta diversity revealed a different microbiota profile between the two groups, indicating an altered microbial community structure in the ASD group. This result was supported by previous studies (Finegold et al., 2010; De Angelis et al., 2013; Strati et al., 2017; Kang et al., 2018; Liu et al., 2019). The decreased gut microbial diversity in children with ASD was concordant with studies by Kang et al. (2013, 2018) and Liu et al. (2019). In contrast, Son et al. found no visible changes in the diversity and richness of gut microbiota in the stools of subjects with ASD and neurotypical sibling controls (Son et al., 2015). Finegold et al. and Angelis et al. found greater microbial diversity in subjects with ASD than in controls (Finegold et al., 2010; De Angelis et al., 2013). In comparison with the previous studies, the case-control subjects in our research had similar lifestyle characteristics and were matched for sex and age in a narrow age range. In addition, this design had a relatively larger sample size than most of the previous reports which usually involved fewer than 25 participants in each group (Finegold et al., 2010; Wang et al., 2011; Williams et al., 2011; Kang et al., 2013, 2018; De Angelis et al., 2015; Tomova et al., 2015; Rose et al., 2018), thus could provide more robust conclusions. Furthermore, differences in the diagnosis criteria (e.g., the Autism Diagnostic Observation Schedule, the Autism Diagnostic Interview-Revised or the CARS), the methods used to investigate the subject samples (16S rRNA, qPCR, or culture), genetic and/or dietary background, and autism severity (Desbonnet et al., 2014; Wang et al., 2014) might be other potential reasons for the study heterogeneity.

### Autism-Associated Changes in Gut Microflora at Different Levels

Consistent with Kang et al. (2013), we found no significant difference in the relative abundance of microbiota on the level of phylum between the ASD and neurotypical groups. However, previous studies have shown changes in the relative abundance of phylotypes, such as *Firmicutes, Bacteroidetes, Proteobacteria*, and *Actinobacteria* (Williams et al., 2011; De Angelis et al., 2015; Tomova et al., 2015; Strati et al., 2017; Liu et al., 2019). In addition, some studies have previously reported a shift toward a lower proportion of *Bacteroidetes* and a higher level of *Firmicutes* for fecal samples of ASD children (Williams et al., 2011; Tomova et al., 2015; Strati et al., 2017). Angelis et al., in contrast, found the opposite result (De Angelis et al., 2015). Family level analysis in our data showed a significant reduction of *Acidaminococcaceae* in the fecal samples of ASD subjects compared to the healthy controls, which had not been reported previously.

Comparing the microbial taxa at the genus level between the ASD and healthy groups, *Flavonifractor* (family *Ruminococcaceae*), *Lachnoclostridium, Tyzzerella subgroup 4*, and *unidentified Lachnospiraceae* (family *Lachnospiraceae*) were significantly lower in children with ASD in the present study. Liu et al. also found a signifcant decrease of *Lachnospiraceae NC2004 group* (family *Lachnospiraceae*) in ASD group (Liu et al., 2019). In contrast, based on 21 autistic and 7 typically developing samples with GI symptoms, Rose et al. found that children with ASD showed more *Lachnospiraceae* than typically developing groups (Rose et al., 2018). De Angelis et al. demonstrated that members in the *Lachnospiraceae* family either increased or decreased in children with ASD compared to healthy controls (De Angelis et al., 2013). Increasing evidence from animal studies supports the hypothesis that intestinal microbiota have evolved to exert a marked influence on the central nervous system function via inflammation, and the hypothalamic–pituitary–adrenal axis, by affecting neurotransmission. Inflammatory activity, assessed by proinflammatory cytokines, such as tumor necrosis factor alpha, interleukin 1β, and interleukin 8, were enhanced in children with ASD (Tonhajzerova et al., 2015). Members of the *Lachnospiraceae* and *Ruminococcaceae* family are butyrate producers (Meehan and Beiko, 2014). Butyrate production in the human gut is highly relevant because it promotes Treg cell differentiation, which can ultimately suppress proinflammatory responses (Singh et al., 2014). A recent study using a mice model with inflammatory bowel disease showed that butyrate can protect the integrity of intestinal epithelial barrier, which can reduce the inflammatory response in inflammatory bowel disease such as Crohn's disease (Chen et al., 2018). In addition, butyrate can regulate synthesis of the neurotransmitters dopamine by altering expression of the tyrosine hydroxylase gene (Decastro et al., 2005). However, we found that apart from *Lachnoclostridium* genus, all other significant differences were related to very low-abundant genera (0.08–0.16%). The cross-sectional nature of the study did not enable us to understand the mechanisms and time sequence of the associations. New studies that incorporate repeated, prospectively collected fecal samples will be important to elucidate whether those bacteria with low abundance could really result from or have an influence on the disease in ASD patients.

A recent systematic review demonstrated an interrelation between Clostridium bacteria colonization of the intestinal tract and autism (Argou-Cardozo and Zeidan-Chulia, 2018). The species belonging to the *Clostridium* have been shown to produce exotoxins (Stiles et al., 2014) and p-cresol, cause higher propionic acid levels (Larroya-Garc et al., 2018), and promote conditions that favor inflammation that may exacerbate autistic symptoms (Shen, 2012). It may also interacts with beneficial bacteria such as *Bifidobacterium* to play a role in the pathogenesis of ASD (Larroya-Garc et al., 2018). We also observed that *Clostridium clostridioforme*, affiliated to *Clostridium* genera, was higher in the ASD group than in healthy children. Nevertheless, possible neurotoxic effects of bacterial metabolites require further research to assess their exact effect and how they can be altered. Data from India (Pulikkan et al., 2018), Slovakia (Tomova et al., 2015) and the United States (Adams et al., 2011) revealed a significantly high richness of genus *Lactobacillus* in children with ASD. Kang et al. (2013, 2018) and De Angelis et al. (2013) suggested an enrichment of the genus *Prevotella* in healthy subjects compared to autistic samples. However, in this study, we did not detect any significant differences of these bacteria between autistic and healthy children. Potential issues connected to mis-classification at species-level using 16S rRNA gene method could not be excluded in the present study.

In our data, substantial covariate information collection was allowed for adjustment of potential confounders in the analysis of group differences at different levels of microbiome whereas most of the previous used univariate analysis to draw conclusion (Finegold et al., 2010; Adams et al., 2011; Wang et al., 2011, 2013; Williams et al., 2011; De Angelis et al., 2013; Tomova et al., 2015; Strati et al., 2017; Kang et al., 2018; Pulikkan et al., 2018; Zhang et al., 2018; Liu et al., 2019). We also applied the FDR to correct multiple comparisons which has not been considered in some of the previous studies (Finegold et al., 2010; Wang et al., 2011, 2013; Williams et al., 2011; De Angelis et al., 2013). Therefore, covariates adjustment and inadequate statistical control for testing multiple hypotheses might also provide possible explanations for the different findings at each level of bacteria across studies in addition to the potential reasons mentioned above (Desbonnet et al., 2014; Wang et al., 2014).

No significant functional differences were found between groups after FDR correction, which was consistent with Kang et al. using the PICRUSt to estimate metabolic function (Kang et al., 2018). Rose et al. reported that the pathways correlated to the *two components system* were under-represented in ASD children compared with healthy controls (Rose et al., 2018). However, the functional differences based on 16S rRNA gene method relies on an open but incomplete reference genome database, thus predictions should be interpreted with caution (Langille et al., 2013; Asshauer et al., 2015). Future studies with microbial metagenomic sequencing analysis should be carried out to obtain information about the functional diversity of the bacterial community.

## Conclusions

In conclusion, we found that children with ASD had lower quantities of *Acidaminococcaceae*, genera *Flavonifractor, Lachnoclostridium, Tyzzerella subgroup 4*, and *unidentified Lachnospiraceae* and an elevated proportion of *Clostridium clostridioforme* compared to neurotypical controls.

## Data Availability

The datasets for this manuscript are not publicly available because of limitations due to participant consent. Requests to access the datasets should be directed to Jin Jing, jingjin@mail.sysu.edu.cn.

## Ethics Statement

The Ethical Committee of the School of Public Health, Sun Yat-sen University approved this study (NO.2018047). The study was performed in accordance with ethical principles in the Declaration of Helsinki, including that the subjects were volunteers and that them and their legally authorized representatives were adequately informed of the aims, methods, the anticipated benefits and the potential risks of the study and the discomfort it might entail. All subjects were informed of the sources of funding and the possible conflicts of interest and the institutional affiliations of the researchers. Every precaution was taken in order to grant the privacy of the research subjects (World Medical Association, 2013). The parents or guardians of all subjects provided their written informed consent before the study.

## Author Contributions

JJ and ZZ conceived the study and designed the experiments. JJL, MD, and JW recruited subjects and collected specimens. BM and JYL performed the experiments and analyzed the data. BM and JJL wrote the manuscript. JJ and ZZ revised the manuscript. All the authors critically reviewed and approved the manuscript.

### Conflict of Interest Statement

The authors declare that the research was conducted in the absence of any commercial or financial relationships that could be construed as a potential conflict of interest.

## Abbreviations

ASD: Autism Spectrum Disorder
NT: neurotypical
16S rRNA: 16S ribosomal RNA
GI: gastrointestinal
DMS-5, Diagnostic and Statistical Manual of Mental Disorders: 5th Edition
CARS: Childhood Autism Rating Scale
OTUs: operational taxonomic units
ACE: abundance-based coverage estimator
PD: phylogenetic diversity
PCoA: principal coordinates analysis
PERMANOVA: permutational multivariate analysis of variance
FDR
false discovery rate.
