# Supplementary material for: Altered Gut Microbiota in Chinese Children With Autism Spectrum Disorders
Source: Front Cell Infect Microbiol. 2019 Mar 6;9:40. doi: 10.3389/fcimb.2019.00040 (PMC6414714; doi:10.3389/fcimb.2019.00040)
Supplement: Supplementary file 1 [file Data_Sheet_1.docx]

***Supplementary Material***

**Altered Gut Microbiota in Chinese Children with Autism Spectrum Disorders**

**Bingjie Ma^#^, Jingjing Liang^#^, Meixia Dai, Jue Wang, Jingyin Luo, Zheqing Zhang^*^, Jin Jing^*^**

***Correspondence:**

**Jin Jing**, PhD, MD

E-mail: [jingjin@mail.sysu.edu.cn](mailto:jingjin@mail.sysu.edu.cn)

**Zheqing Zhang**, PhD, MD

Email: zzqaa501@smu.edu.cn

**^#^ Bingjie Ma and Jingjing Liang contributed equally to this article.**

1. **Supplementary Figures and Tables**
   1. **Supplementary Tables**

Supplemental Table S1. Sequencing data summary with the number of a total sequence reads, qualified sequence reads and OTUs.

Supplemental Table S2. Alpha diversity indices of bacterial phylotypes between NT and ASD groups.

Supplemental Table S3. PERMANOVA tests of the bacterial gut microbiota on the Bray-Curtis dissimilarity, unweighted and weighted UniFrac distances according to subjects’ health status.

Supplemental Table S4. Relative abundance of 10 phyla detected in NT and ASD groups.

Supplemental Table S5. Relative abundance of top 10 abundant classes detected in NT and ASD groups and class presenting significant difference between NT and ASD groups.

Supplemental Table S6. Relative abundance of top 10 abundant orders detected in NT and ASD groups and order presenting significant difference between NT and ASD groups.

Supplemental Table S7. Species presenting significant difference between NT and ASD groups.

Supplemental Table S8. Tax4Fun showing predicted relative abundance of KEGG ortholog groups (Level 2 KOs).

- 1. **Supplementary Figures**

**
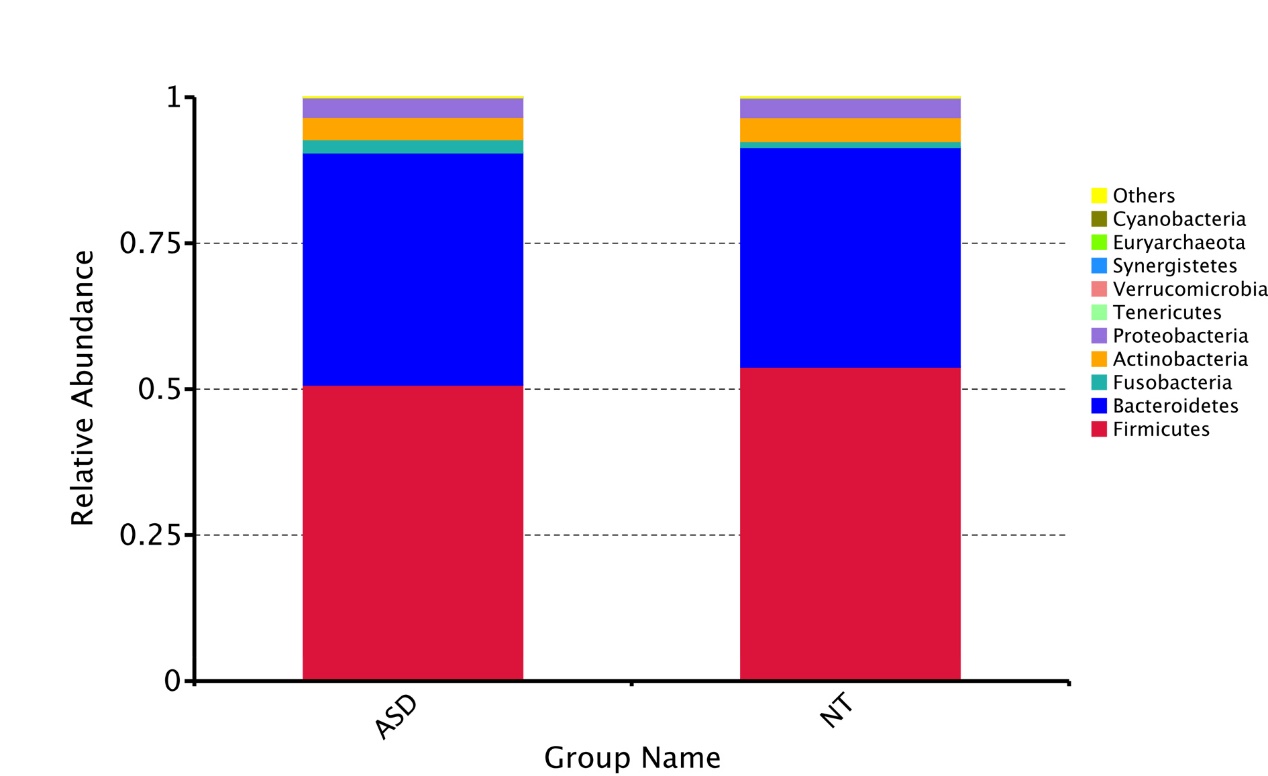
**

Supplemental Figure S1. Top 10 abundant phyla in ASD and NT groups. ASD: autism spectrum disorders; NT: neurotypical.


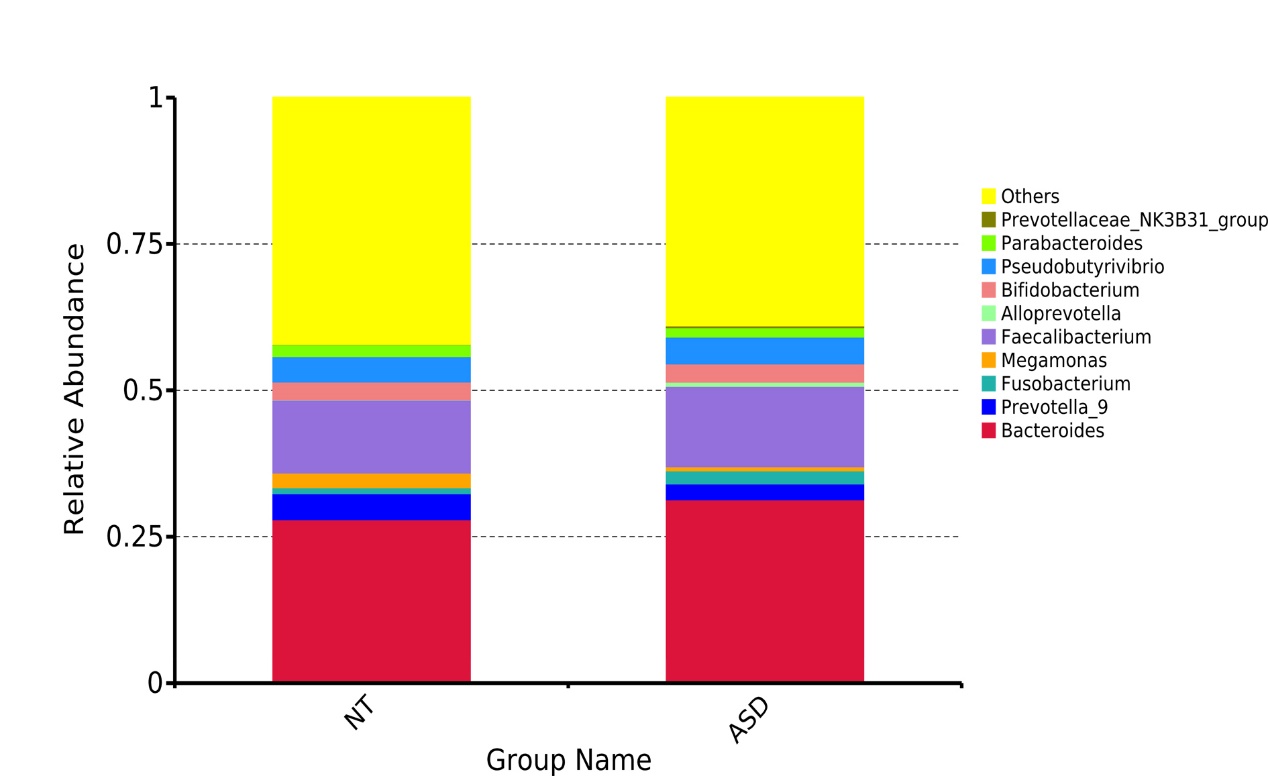


Supplemental Figure S2. Top 10 abundant genera in ASD and NT groups. ASD: autism spectrum disorders; NT: neurotypical.
